# Development of an age-dependent cognitive index: relationship between impaired learning and disturbances in circadian timekeeping

**DOI:** 10.3389/fnagi.2022.991833

**Published:** 2022-11-09

**Authors:** Karienn A. Souza, Andrew Powell, Gregg C. Allen, David J. Earnest

**Affiliations:** ^1^Department of Neuroscience and Experimental Therapeutics, School of Medicine, Texas A&M Health Science Center, Texas A&M University, Bryan, TX, United States; ^2^Center for Biological Clocks Research, Texas A&M University, College Station, TX, United States

**Keywords:** Barnes maze, entrainment, activity rhythm, aging, middle aged, mice, cognitive index

## Abstract

Preclinical quantitative models of cognitive performance are necessary for translation from basic research to clinical studies. In rodents, non-cognitive factors are a potential influence on testing outcome and high variability in behavior requires multiple time point testing for better assessment of performance in more sophisticated tests. Thus, these models have limited translational value as most human cognitive tests characterize cognition using single digit scales to distinguish between impaired and unimpaired function. To address these limitations, we developed a cognitive index for learning based on previously described scores for strategies used by mice to escape the Barnes maze. We compared the cognitive index and circadian patterns of light-dark entrainment in young (4–6 months), middle-aged (13–14 months), and aged (18–24 months) mice as cognitive changes during aging are often accompanied by pronounced changes in sleep-wake cycle. Following continuous analysis of circadian wheel-running activity (30–40 days), the same cohorts of mice were tested in the Barnes maze. Aged mice showed significant deficits in the learning and memory portions of the Barnes maze relative to young and middle-aged animals, and the cognitive index was positively correlated to the memory portion of the task (probe) in all groups. Significant age-related alterations in circadian entrainment of the activity rhythm were observed in the middle-aged and aged cohorts. In middle-aged mice, the delayed phase angle of entrainment and increased variability in the daily onsets of activity preceded learning and memory deficits observed in aged animals. Interestingly, learning-impaired mice were distinguished by a positive relationship between the extent of Barnes-related cognitive impairment and variability in daily onsets of circadian activity. While it is unclear whether changes in the sleep-wake cycle or other circadian rhythms play a role in cognitive impairment during aging, our results suggest that circadian rhythm perturbations or misalignment may nevertheless provide an early predictor of age-related cognitive decline.

## Introduction

Cognitive decline is the clinical hallmark of age-related dementia and Alzheimer’s disease (AD). The hippocampus, the region of the brain responsible for spatial memory (Gallagher, 1997; Foster et al., 2012), is severely affected in AD, and behavioral assessments which can probe the integrity of this region have been historically used in diagnosis. However, most patients are in late stages of the disease when they receive this diagnosis. With continued characterization of age-related dementia, it has become clear that the hallmark cognitive decline is often preceded by atypical behavioral symptoms, including pronounced alterations in circadian rhythms, and that this combination of functional impairments reflect degeneration in multiple systems. Thus, the development of preclinical models to identify specific behavioral changes that occur early in the progression of age-related cognitive impairment could yield a better index and advance diagnosis of dementia.

Rodents and humans share correlative similarities between brain structures and their functions in learning and memory. As such, aged rodent models have been useful in preclinical studies, exhibiting similar hippocampal deficits and performance variability to those observed in humans (Gallagher and Rapp, 1997). More specifically, during normal aging, cognitive decline is observed in part of the aged population, while others may show no significant cognitive change and perform within the range of their younger counterparts. To characterize behavior and improve translation of findings from these animal models to humans, Gallagher et al. (1993) developed the Spatial Learning Index (SLI) for the water maze. This quantitative index scores animals based on their hippocampal performance and allows for individual variability to be represented within study cohorts, thereby facilitating the separation of better learners from learning-impaired in the aged cohort (Tomás Pereira and Burwell, 2015). Although the water maze has significant utility in behavioral studies, there are limitations with its use as a cognitive assessment in mice (Harrison et al., 2009). For example, some mouse models show low motivation for swimming and searching for an escape in water (Whishaw and Tomie, 1996). In addition, the water maze may not be ideal for rodent models involving significant motor deficits, such as models of spinal cord or traumatic brain injury and stroke (Leconte et al., 2020; Panta et al., 2020). The Barnes maze is also less stressful than the water maze and animals may habituate quicker to the maze requiring less trials to reach ceiling performance (Harrison et al., 2009). Thus, the development of similar indices for other tasks is necessary for the advancement of research on age-related changes in cognitive function.

The Barnes maze represents a suitable alternative for the water maze. There are many variations of this task, but in general, there is a learning phase in which mice learn how to find the escape route and a probe trial (or trials) in which memory of the escape position can be assessed (Barnes, 1979; O’Leary and Brown, 2013). Here we focus on the development of a single cognitive index for the learning phase of the Barnes, based on strategy scoring first devised by Illouz et al. (2016). Multiple variables can be assessed during this task for cognitive (learning and memory of spatial-dependent navigation) and non-cognitive behaviors (speed, mobility). Distance and latency have been often used to demonstrate proficiency in finding the escape (Pitts, 2018; Rees et al., 2020). Quantification of latency may show level of efficiency to find the goal, but this variable is affected by age-dependent speed differences (Bizon et al., 2009). Although these measures are useful for determining group differences, distance and speed do not consider the efficacy of the different strategies that mice use to find the escape hole, and thus the aforementioned parameters only provide limited information on cognitive capacity. Rodents use multiple approaches to navigate the Barnes maze, including non-hippocampal strategies, such as serial search, that often result in large distances as mice search systematically until reaching the goal. By identifying different navigation strategies with validated scores, we are able to quantify the extent of efficiency on the learning acquisition without relying solely in hippocampal strategies (National Sleep Foundation, 2003; Illouz et al., 2016). Furthermore, quantification of performance with single numerical value allows for direct comparisons with other biomarkers, and also for within group comparisons to detect impairment in aging rodent models. Using the validated scores for each type of strategy, we created a single summed cognitive index for the learning phase of the task to evaluate Barnes performance across the aging spectrum of C57Bl/6 mice. Additionally, the cognitive index from the learning phase of the Barnes task was correlated with the probe (memory) trial, showing that this score provides a useful descriptor of cognitive performance even if its derivation is not solely from hippocampal-dependent performance.

To gauge the potential of the cognitive index as an indicator and/or predictor of cognitive impairment during aging, the same cohort of mice was analyzed for parallel changes in the circadian regulation of sleep-wake rhythms, especially with regard to their entrainment to the light-dark cycle. The prevalence of sleep disorders, especially those affecting the circadian regulation of the sleep-wake cycle, increases with advancing age (National Sleep Foundation, 2003). These sleep disorders and circadian rhythm disturbances occur during healthy aging but some are distinctive and more pronounced in age-related dementia and AD (Homolak et al., 2018). In contrast to the circadian and sleep-wake disturbances typically observed in healthy elderly individuals, delayed sleep-wake patterns in which both bedtime and wake times occur later in the day and irregular sleep-wake rhythm disorder with high variability in the timing of sleep onset and wakefulness are commonly observed in patients with mild to severe dementia (Wang et al., 2015; Canevelli et al., 2016; Homolak et al., 2018; Leng et al., 2019). Based on the link between distinctive circadian rhythm alterations and cognitive impairment in aging (Devan et al., 2001; Craig and McDonald, 2008) and AD (Wulff et al., 2010), we explored the relationship between changes in cognition and light-dark entrainment of the circadian activity rhythm across the lifespan of the C57Bl/6 mouse model. Marked alterations in the light-dark entrainment of the activity rhythm were observed early in middle-aged mice prior to any age-related deficits in Barnes maze performance, demonstrating their potential predictive value for further cognitive decline. The correlation between the cognitive index of learning-impaired mice in Barnes maze and circadian variables suggests that there may be a relationship between cognition deficits and circadian disturbances across all age groups. Our cognitive index, used in combination with correlative changes in circadian behavior, thus offers a putative model for early diagnostic and detection of age-related cognitive impairment.

## Materials and Methods

### Animals

Wild type male and female C57Bl/6 were obtained from a breeding colony from breeding pairs purchased from the Jackson Laboratory (stock #014548). The breeding colony was maintained by the Comparative Medicine Program at Texas A&M and new breeder pairs were purchased and introduced accordingly as per Jackson Laboratory’s protocols for breeding. Based on comparisons with our published data (Bang et al., 2021), there were no signs of any strain variability or variability in behavioral and physiological measures due to crossbreeding. All mice were maintained under controlled conditions (22°C–25°C) on a standard 12 h light:12 h dark cycle (LD 12:12; lights-on at 0700 h) in the AAALAC-accredited vivarium at the Texas A&M University Health Science Center. All animal experiments were performed in accordance with the National Institutes of Health Guide for the Care and Use of Laboratory Animals. Animal procedures used in this study were conducted in compliance with Animal Use Protocol 2019-0265 as reviewed and approved by the Institutional Animal Care and Use Committee at Texas A&M University.

**Figure 1:**
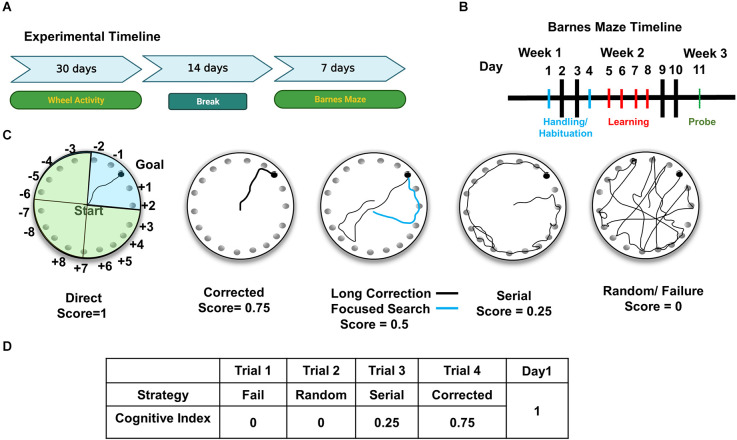
Experimental design for cognitive and circadian behavior analysis. **(A)** For the experimental timeline, mice were first analyzed for circadian wheel-running activity, followed by Barnes maze testing. **(B)** Barnes maze testing consisted of 7 days of behavioral analysis, including habituation to the apparatus in days 1 and 2, training/learning of the position of escape hole (days 3–6), and a 72 h probe on day 7. **(C)** Schematic representation of the Barnes maze shows faux escape boxes (numbered) and goal location (escape box). For illustration purposes, maze is divided into four quadrants. Blue shaded quadrant is the target quadrant used for Probe analysis. Representative strategies are shown in each circular depiction of the Barnes. Spatial strategies include direct, corrected, long correction (black trace), focused search (blue trace). **(D)** Example of scoring of trials in Day 1 for a mouse. Cognitive indices are calculated per mouse by adding the scores from each trial for all 4 days.

Mice used for analysis of the circadian rhythm of wheel-running behavior were: young (4–6 months, *n* = 21, 12 males and nine females), middle-aged (13–14 months, *n* = 20, nine males and 11 females) and aged (18–22 months, *n* = 29, 14 males and 15 females). These mice were housed individually in cages equipped with running wheels to provide for continuous analysis of wheel-running activity during the analysis of circadian behavior, which lasted 30 days. Two weeks after the analysis of circadian behavior, mice were habituated and tested in the Barnes maze (Figure 1A). Mice used for Barnes maze analysis were: young (*n* = 21, 12 males and nine females), middle-aged (*n* = 21, 10 males and 11 females) and aged (*n* = 29, 14 males and 15 females). Only mice with values for both circadian and Barnes behavior analyses were used in the correlational comparisons (shown in Figure 5).

**Figure 2:**
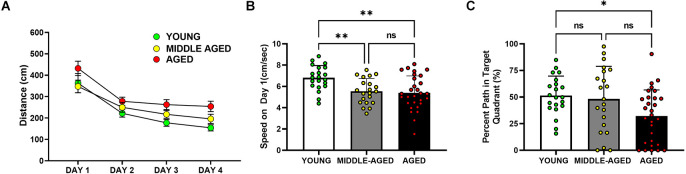
Cognitive performance of aged mice (18–22 months) is impaired in the Barnes maze, but not in middle-aged mice (13–14 months) relative to the young cohort (4–6 months). **(A)** Analysis of the distance traveled by mice to locate the escape box. Aged mice showed longer distance traveled to the escape than young and middle-aged mice (*), such that distance traveled in young and middle-aged mice was significantly decreased in comparison with the values observed in aged mice (*, *post hoc* Tukey/Kramer, *p* < 0.05). **(B)** Speed on the first day of Barnes performance, pre training. Young mice moved faster than the middle-aged and aged cohorts (**, *post hoc* Tukey’s multiple comparison, *p* < 0.01; middle-aged vs. aged, ns = non-significant, *p* > 0.05). **(C)** Percent of the path localized in the target quadrant (i.e., quadrant in which the escape was localized during training trials, during the first 30 s of the trial). Only the young and aged groups are marked by significant differences (*, Tukey’s multiple comparison; *p* < 0.05). In **(B)** and **(C)**, bars depict Mean ± SEM values of the corresponding age group. Circles indicate individual scores of each mouse.

**Figure 3:**
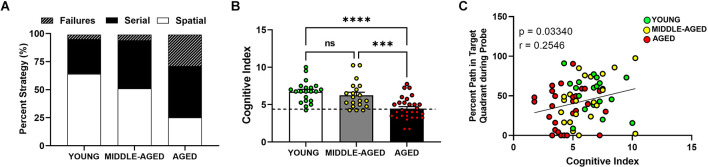
Aged mice (18–22 months) show lower hippocampal-dependent performance in Barnes maze. **(A)** Representative between-group comparison of profiles of strategy selection by each age group for entire training session (prior to probe). **(B)** Summed cognitive index comparison for training trials showed an age effect due to lower use of spatial strategies. Mean ± SEM value of the corresponding age group is shown and dashed line represents the cut off for division between high and low performers (i.e., learning-impaired mice). Young and middle-aged cohorts are not different, but aged mice have significant lower scores (Tukey’s multiple comparison; young vs. aged, ****p < 0.0001; middle-aged vs. aged; ***p < 0.001; young vs. middle-aged, ns = non-significant, *p* > 0.05). **(C)** Correlation between cognitive index and probe analysis of Barnes performance. Circles indicate individual scores of each mouse and are color coded as in **(B)**. Cognitive index was positively correlated with the percent path values from the probe trial (Person correlation *p* < 0.05).

**Figure 4:**
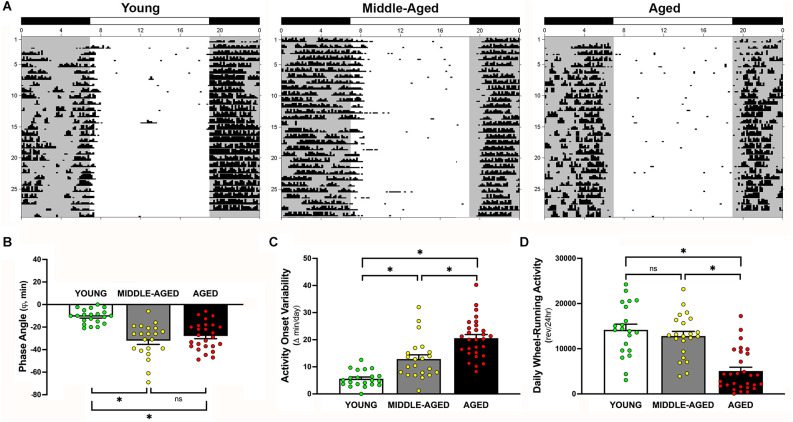
Effects of aging on light-dark entrainment and other properties of the circadian rhythm of wheel-running activity in mice. **(A)** Representative records of circadian wheel-running behavior in young (left), middle-aged (center), and aged (right) mice during entrainment to LD 12:12. Actograms are plotted over a 24-h period. The closed bars at the top and shading on the records signify the timing of the dark phase in the LD 12:12 cycle. Comparisons of **(B)** the phase angle (φ) between daily activity onsets and lights-off, **(C)** absolute day-to-day variability, and **(D)** total daily wheel-running activity (wheel revolutions/24 h) in young, middle-aged, and aged mice during entrainment to LD 12:12. In panel **(B)**, negative phase angle values (in minutes) indicate that daily onsets of activity occur after lights-off whereas positive values denote that activity onsets precede the end of the light phase. Bars (in **B–D**) depict Mean ± SEM values in young, middle-aged, and aged mice. Circles indicate individual scores of each mouse (*, Tukey’s multiple comparison; *p* < 0.0001; young vs. aged; middle-aged vs. aged; young vs. middle-aged, ns = non significant, *p* > 0.05).

**Figure 5:**
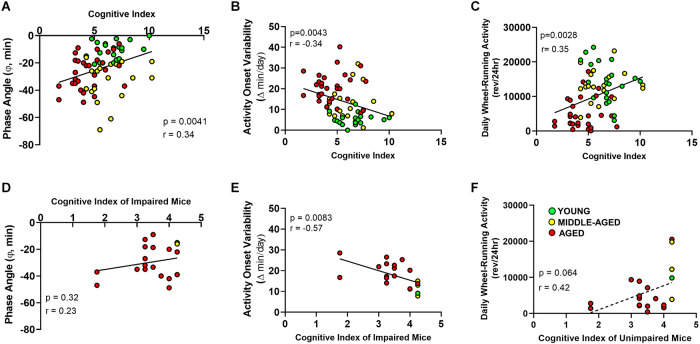
Relationship between cognitive index acquired from Barnes maze and circadian entrainment properties of the activity rhythm. **(A)** The phase angle (φ) between daily activity onsets and lights-off; **(B)** absolute day-to-day variability in onsets of activity; and **(C)** total daily wheel-running activity (wheel revolutions/24 h) are correlated with Cognitive Index for young, middle-aged, and aged mice. Panels **(D–F)** show the same circadian measures as in **(A–C)**, however only for learning-impaired. Circles indicate individual scores of each mouse (young *n* = 1, middle-aged *n* = 3 and aged *n* = 16). Activity onset variability, but not phase angle of entrainment or total wheel-running activity, of learning-impaired mice are negatively correlated (*r* = −0.57) with cognitive index.

### Barnes maze

The Barnes circular platform maze is a 66 cm diameter circular platform on a 1.4 m stand with 20 evenly spaced 5.08 cm diameter holes around the circumference, where a black box (escape tunnel) was placed underneath one of the holes (San Diego Instruments, San Diego, CA, USA). Four bright lights were positioned above the maze as an aversive stimulus. Between each trial, the platform and escape tunnel were cleaned with 70% ethanol and water, and video was acquired using a Color GigE camera (model: acA1300-30gc). Data were quantified using Ethovision XT 16 video tracking software (Noldus, Leesburg, VA, USA).

The protocol consists of habituation, acquisition training (learning) and probe (Figure 1B; Rees et al., 2020).

#### Habituation

During habituation day 1, mice were placed on the table for 5 min and allowed to explore the maze without an escape box, under dim lighting. In day 2, mice were placed in a 2 L transparent glass beaker under aversive lighting, and then after 1 min were gently guided to the escape hole and lights were turned off.

#### Learning trials

In all subsequent days (3–6), the mice were individually placed in the center of the table under a dark container for 30 s before the container was lifted and the mouse was allowed to navigate the maze. Each mouse was allowed 180 s to locate and enter the escape box per trial. If the mouse was unable to locate the escape hole after 180 s, it was gently guided to the correct hole location and allowed to enter the escape tunnel. Once the mouse entered the escape tunnel (either guided or on their own), it remained in the tunnel for 1 min before returning to its home cage. Each day, the mice received four trials spaced 15 min apart for a total of 16 learning training trials over 4 days.

#### Probe trial

A probe trial was done 72 h after the last day of the learning phase. During this single trial, on day 7, mice were placed on the table as described for the learning trials, but the escape box was not available. After 180 s, the mouse was removed and placed back into their holding cage.

#### Search strategies and development of the cognitive index

In order to analyze Barnes maze performance, each trial was scored using a modified scale created by Illouz et al. (2016). From left to right, representative paths of the possible strategies that a mouse can choose to find the escape are shown in Figure 1C. The hippocampal-dependent strategies are: direct (no error; score = 1), corrected (searched + or – 1 immediate hole, score = 0.75), focused (searched + or – 3 immediate holes, score = 0.5), and long correction (mouse searches across the target and immediately corrects toward correct hole, score = 0.5). Non-hippocampal strategies include the serial search (animal methodically searches holes one by one, score = 0.25), random (search without a clear strategy and target hole identified by chance, score = 0), and failure (animal searches but does not find the target, score = 0). Trials are scored by a blinded investigator. Each trial receives a single score, and scores are summed for each day as shown in the example in Figure 1D. The cognitive index was created by summing the scores of all 4 days.

### Wheel-running activity

The circadian rhythm of wheel-running activity was continuously recorded for at least 30 days. Wheel-running activity was stored in 10-min bins, graphically depicted in actograms, and analyzed using ClockLab data collection and analysis software (ActiMetrics, Evanston, IL, USA). Entrainment and qualitative parameters of the activity rhythm were measured over the same interval for all animals. During entrainment to LD 12:12, the onset of activity for a given cycle was identified as the first bin during which an animal attained 10% of peak running-wheel revolutions (i.e., intensity). To measure phase angle of entrainment (φ), least squares analyses was used to establish a regression line through the daily onsets of activity during the period of entrainment (30 days), and then the number of minutes before (positive) or after (negative) the time of lights-off in the LD cycle (1,800 h) was determined for each animal. Total daily activity was calculated by averaging the number of wheel revolutions per 24 h over the 30-day interval of analysis.

### Statistical analysis

In each case, differences in behavior were considered significant at *p* < 0.05 (GraphPad, San Diego, CA, USA). Statistical analyses were performed on the raw data using one or two-way (age alone, or age and sex) repeated measures ANOVA to determine the significance of age-related differences when the data was analyzed across days for significance. One-way ANOVAs were performed on all other comparisons.

For probe trials, the percent of the time in the target quadrant is used to quantify the animal’s insistence about the escape hole’s location. Quadrants contain five possible locations for escape, and the escape hole is in the middle of the target quadrant (blue shaded quadrant in Figure 1C). Only data from the first 30 s are analyzed because mice typically give up searching after approximately 30 s. Group means from the probe trial were analyzed with one-way ANOVAs. Newman-Keuls* post hoc* analysis were applied when necessary to determine the significance of age-related differences in circadian entrainment and quantitative parameters of the activity rhythm. Tukey/Kramer (or Tukey’s for multiple comparisons) *post hoc* analysis was used for more comprehensive Barnes maze analysis. Correlations were analyzed by Spearman’s correlation with significance *p* < 0.05.

## Results

### Aged mice, but not middle-aged, are impaired in the Barnes maze

Many Barnes maze measures can be used to measure learning and memory. In our analysis of distance traveled to the target (Figure 2A), aged mice took longer paths (distance in cm) to find the escape box than the middle-aged and young mice (*F*_(2,65)_ = 8.903, *p* < 0.001). *Post-hoc* analysis of distance traveled further demonstrated that the aged group was statistically different from young (*p* < 0.01) and also from middle-aged cohorts (*p* < 0.01), with no difference observed between young and middle-aged mice (*p* = 0.27). Mice of all ages performed better across time (*F*_(3,195)_ = 32.893, *p* < 0.0001), with all age groups demonstrating less distance to reach the goal across days. There was no overall effect of sex in the distance measure (*F*_(1,65)_ = 0.109, *p* = 0.74). Speed changes as the animals learn the position of the platform and can vary depending on the strategy. On the first day when mice are naïve and untrained, the average speed was significantly different (*F*_(2,68)_ = 7.401, *p* < 0.001) between young, middle-aged, and aged mice (Figure 2B). The young cohort moved around faster than middle-aged and aged mice. Speed on day 1 did not differ between the middle-aged and aged groups but was significantly decreased (Tukey’s multiple comparison; *p* < 0.01) in comparison with the young cohort. We did not observe a sex difference on speed on day 1 (*F*_(1,68)_ = 3.219, *p* = 0.077).

During the first 30 s of the probe trial, shown in Figure 2C, when the escape box was not available, we tested whether mice used their learning experience and concentrated their search in the target quadrant. Age was a significant factor in this measure of memory (*F*_(2,68)_ = 4.425, *p* < 0.05). Young and aged mice were the only group comparison with significant differences (Tukey’s multiple comparison; *p* < 0.05) in this measure, such that the percent of path in the target quadrant was greater in young animals than in the aged cohort. There was no effect of sex on this measure (*F*_(1,68)_ = 1.050, *p* = 0.31).

### Aged mice show more failures and less preference for spatial strategies

Hippocampal-dependent performance was observed to change as mice aged (Figure 3A). Aged mice preferred nonspatial (failures or serial) strategies (about 50% of the time) to spatial (about 25%) and showed more failures (25%) than their middle-aged and young (about 5%) counterparts. We then summed the cognitive scores for each animal for all learning days and compared the mean cognitive index scores for each group with a one-way ANOVA (Figure 3B). Higher scores indicate that spatial strategies were used more often than serial strategies and that fewer failures were detected. Age had a significant effect on escape strategy preference (*F*_(2,68)_ = 14.66, *p* < 0.0001), but the difference was only due to the aged group as the young and middle-aged cohorts were characterized by similar cognitive indexes. Both the young and middle-aged groups had significantly higher (Tukey’s multiple comparison; *p* < 0.001) cognitive indices than aged mice. No effect of sex was observed in the cognitive index measure (*F*_(1,68)_ = 0.12, *p* = 0.73). Young and middle-aged groups showed similar range and variability within their cognitive indices, ranging from 4.25 to 10, whereas the scores in aged mice were lower and ranged between 1.75 and 7.75. The minimum CI score was 4.25, representing the lowest performer in the young cohort. To further evaluate the relationship between cognitive index scores and other variables, we used this score as a differential to specifically identify and separate low performers (learning-impaired) on this task in each age cohort. The dashed line in Figure 3B delineates this separation of learning-impaired (at CI = 4.25).

### Cognitive score from learning trials correlates with memory score from probe

Further analysis using Pearson correlations revealed a significant positive correlation between the cognitive index and the percent path values from the probe trial (Figure 3C; Pearson correlation *p* < 0.05, *r* = 0.25). Based on comparisons within individual age cohorts (data not shown separately), there was no correlation between cognitive index and this variable in the young (Pearson correlation, *p* = 0.32, *r* = 0.053), middle aged (Pearson correlation, *p* = 0.17, *r* = 0.32), or aged (Pearson correlation, *p* = 0.58, *r* = 0.107) mice. The lack of correlation within individual age cohorts provides evidence that the overall correlation is age-dependent. The overall correlation also is consistent with age-related differences in the distance data because the effect of age is the main driver of the changes in cognitive performance. This relationship further confirms that the cognitive index is a reliable score to grade the level of cognition (spatial/hippocampal) during the learning or training phase of the Barnes maze.

### Altered entrainment of the circadian rhythm activity occurs as early as middle-age and persists through aging

During exposure to LD 12:12, entrainment of the activity rhythm was observed in all young, middle-aged, and aged mice. Representative actograms of mice from each age group are shown in Figure 4A. Comparisons of young mice with the middle-aged and aged cohorts revealed clear differences in their patterns of circadian entrainment (overall effect of age; *F*_(2,69)_ = 19.67, *p* < 0.0001). In young mice, their daily onsets of activity occurred shortly after lights-off such that the average phase angle (φ) between the daily onset of activity and the offset of the photoperiod was −10.7 ± 1.4 min (Figure 4B). In contrast, the activity rhythms of middle-aged and aged mice were distinguished by an altered phase angle of entrainment to LD 12:12 such that their daily onsets of activity were delayed and occurred at later times relative to young animals, commencing up to 30–70 min after lights-off for some animals. The average values for φ between activity onsets and lights-off in middle-aged mice (−32.6 ± 3.4 min) and in aged animals (−27.5 ± 2.2 min) were significantly different (*p* < 0.001) from that observed in young mice. However, no significant difference in φ was observed between middle-aged and aged groups (*p* = 0.46). In conjunction with the delayed onsets of daily activity, middle-aged and aged mice showed unstable patterns of entrainment to LD 12:12 in which the timing of their activity onsets was highly variable between successive days (effect of age; *F*_(2,69)_ = 34.13, *p* < 0.0001). During exposure to LD 12:12, the activity onsets in individual middle-aged and aged mice occurred at (earlier or later) times that differed on average by 12.9 min and 20.6 min, respectively from the preceding day, whereas the average day-to-day variability in activity onset times of young animals was only 5.6 min (Figure 4C). The absolute day-to-day variation in the onsets of activity in middle-aged and aged animals were significantly greater (*p* < 0.001 and *p* < 0.0001, respectively) than that observed in young mice. Daily activity onset variability was also significantly greater in aged mice than in the middle-aged cohort (*p* < 0.001). Coupled with the differences in φ and the variability in the daily onsets of activity, young, middle-aged, and aged mice were characterized by marked disparities in the total amount of daily wheel-running activity (overall effect of age; *F*_(2,69)_ = 24.58, *p* < 0.0001). During entrainment to LD 12:12, daily activity levels (wheel revolutions/24 h) in young mice and middle-aged were not different from each other (*p* = 0.65) but were significantly (*p* < 0.0001) and approximately 280% and 250% greater (Figure 4D) than in aged mice, respectively.

### Relationship between entrainment activity and cognition

Next, we examined the relationship between changes in cognition and circadian entrainment of the activity rhythm. In this regard, Pearson correlations were used to compare the cognitive index based on the escape strategies used by mice during training trials with three parameters of the activity rhythm characterized by changes during aging: variability in the daily onsets of activity, pattern of light-dark entrainment, and daily activity levels. The phase angle of circadian entrainment to LD 12:12 was correlated with cognitive index when all ages were analyzed as a group (Figure 5A, Pearson correlation, *p* < 0.01, *r* = 0.3384). When learning-impaired mice were analyzed separately, no correlation between CI and phase angle (Figure 5D, Pearson correlation, *p* = 0.32, *r* = 0.23) was observed. The complement group of unimpaired mice with better CI scores also did not show a correlation between the two variables (Pearson correlation, *p* = 0.071, *r* = 0.26).

For young, middle-aged and aged mice, the day-to-day variability in daily onsets of activity was negatively correlated with the Barnes maze cognitive index (Figure 5B; Pearson correlation, *p* < 0.01, *r* = −0.340). When we analyzed only learning-impaired mice, the variability in the daily onsets of activity was significantly and negatively correlated with the cognitive index (Pearson correlation, *p* < 0.01, *r* = −0.57). In this comparison, when activity onset variability was lower, the scores were higher and thus performance was better in the Barnes maze (Figure 5E). The unimpaired mice with better CI performance scores did not show a correlation between these measures (data not shown; Pearson correlation *p* = 0.2403, *r* = −0.1692). The total daily wheel-running activity was similarly correlated with the cognitive index (Figure 5C, Pearson correlation, *p* < 0.01, *r* = 0.351). A non-significant trend toward a correlation between total daily wheel-running activity and cognitive index was observed in learning-impaired mice (Pearson correlation, *p* = 0.06, *r* = 0.42), but not in unimpaired mice (Pearson correlation, *p* = 0.6770, *r* = 0.06). The significant correlations when all ages are analyzed together, but not separately for individual age cohorts again demonstrates that the observed changes in circadian properties and their relation to cognitive index are age-dependent.

## Conclusion

Quantification of cognitive performance in preclinical studies is necessary in order to achieve higher translational power from basic studies to clinical observations. Learning curves are useful for demonstrating performance and change over time, but the lack of a standardized scoring system for aging mouse models presents some difficulty in comparing behavioral performances across cognitive domains. Thus, new methods of cognitive scoring are necessary for optimizing assessment in rodent models. The new index described in this report was designed to provide information about the cognitive performance of individual animals and also to allow for a better distribution of the cognitive strategies used for learning and remembering the escape box location in the Barnes maze. Similar to the spatial learning index (SLI) for the water maze (Gallagher et al., 1993; LaSarge et al., 2007; Bizon et al., 2009), where aged rodents can be sub-divided into “low performers” and “high performers” groups, our score provides a continuous scale for measuring severity of cognitive impairment in Barnes maze. This subgrouping allowed us to investigate if C57Bl/6 mice with lower cognitive indices also exhibited changes in circadian behavior such as light-dark entrainment of the activity rhythm (Figure 5). Thus, the results of the present study are significant in establishing cognitive index scores that can be used to probe the relationship between cognitive impairment and other age-related biological markers in mice.

The different search strategies used by rodents in the Barnes maze test influence the latency and distance, and these measures may not be interpreted as absolute quantification of spatial learning and memory. Our novel cognitive index standardizes quantification of performance regardless of strategy selection and motor proficiency, creating a way to measure overall cognitive performance in aging, and negates the need for a true hippocampal-dependent measure. The baseline scores defined by Illouz et al. (2016) indeed reflect the animal’s strategy choice based on spatial conceptualization of the Barnes maze on each individual trial. Spatial strategies demonstrating that the rodent is purposefully directed toward the goal, either directly running toward the escape, focusing its search in the proximity of the escape or correcting itself, indicate the animal has established a spatial reference map. As testing continues, we observe that the mice become less sensitive to the noxious stimulus, the drive to escape into the dark box diminishes, and the more naturalistic serial search approach becomes as prominent as the spatial search (or more prominent in some cases; Illouz et al., 2020). Modifications to the protocol, such as minimizing the number of trials, allow for focus on the time period prior to this habituation, but by summing the scores, we automatically normalized the quantification without losing the differences as the higher scores from the spatial strategies drive the difference between groups. After learning the task, spatial memory can be tested using probe trials. We observed the aged mice in our study showed less percent amount of the path in the target quadrant, and we interpret this result as a lower memory or certainty for the location of the goal. The positive relationship between the cognitive index and the probe measure in cohorts of all ages further indicates that the cognitive index reflects the severity of learning impairment regardless of influence of additional non-hippocampal mediated behaviors. Thus, our cognitive index is of value for comparisons between experimental groups in aging studies.

Circadian rhythm alterations and related disturbances of the sleep-wake cycle are commonly observed with aging and often precede or accompany the disease progression in AD (Cipriani et al., 2015; Canevelli et al., 2016), thus providing a potential diagnostic tool for assessment of early onset cognitive impairment. The fragmented sleep patterns precede, and thus are thought to contribute to, the decline in learning and memory performance in AD. In conjunction with other studies characterizing circadian rhythm misalignment in mice during aging (Bang et al., 2021), our results demonstrate that aged mice show irregular sleep-wake patterns similar to those observed in the elderly. As we are interested in modeling age-related changes that can be used as biomarkers of early decline in dementia, we also tested middle-aged C56Bl/6 mice. During entrainment to LD 12:12, the sleep-wake rhythms of middle-aged and aged mice showed delayed onsets of activity commencing at later times (by 20–60 min) relative to the young cohort. In addition to the delayed onsets of daily activity, the sleep-wake patterns of these middle-aged and aged mice were marked by fragmented bouts of activity throughout the night and by unstable patterns of entrainment to LD 12:12 as indicated by high variability in the timing of their activity onsets between successive days.

It is interesting that in the low performers group on the Barnes maze, the cognitive index was correlated with key measures of light-dark entrainment, such that lower cognitive index scores were associated with delayed and more unstable patterns of entrainment. The implications of these altered patterns of circadian entrainment in cognitive impairment during aging are unknown. Because age-related changes in circadian rhythms are not restricted to patients with cognitive decline or dementia, comparisons distinguishing two groups of animals based on cognitive performance (i.e., impaired vs. unimpaired) raises a variety of interesting questions about the mechanism of neurodegeneration during aging. The observed relationship between altered circadian rhythmicity and impaired learning suggests that there is a shared mechanism of neural dysfunction for age-related changes in circadian entrainment and cognition, but perhaps some mice are differentially protected from these changes. Consequently, it is possible that these circadian alterations may be “driving” further age-related deficits in cognition, again illustrating the utility of our model in isolating resilience factors that may differentiate unimpaired from impaired learners. These observed changes in the sleep-wake patterns of middle-aged animals also suggest that the brain circuitry responsible for circadian rhythm entrainment may be impaired earlier in the aging process than areas of the brain that mediate cognition.

In summary, the present results indicate that the cognitive index maintains a strong relationship with the memory portion of the Barnes maze and thus provides a sensitive, efficient, and valid approach to assess age-related cognitive deficits. The paired analysis of Barnes maze-acquired cognitive index and circadian entrainment may provide a valuable model within the context of age-related cognitive decline. Our findings that circadian timekeeping disturbances present before cognitive impairment illustrate how alterations in the sleep-wake cycle might fit into the overall progression of the cognitive decline during aging and even in AD. As such, the altered and unstable patterns of circadian entrainment may be used as an early predictor of age-related cognitive impairment. In addition, this information may lead to the development of potential therapeutic strategies based on the mitigation of sleep-wake cycle disturbances/misalignment to at least slow cognitive impairment during aging and its progression in AD.

## Data Availability Statement

The original contributions presented in the study are included in the article, further inquiries can be directed to the corresponding author.

## Ethics Statement

The animal study was reviewed and approved by Texas A&M University accredited AAALAC committee.

## Author Contributions

KS: conceptualization, formal analysis, data acquisition, analysis and curation, visualization, original writing, review and editing. AP: data acquisition. GA: data acquisition, writing—review and editing. DE: conceptualization of circadian behavior study, data analysis, curation and visualization, original writing—review and editing. All authors contributed to the article and approved the submitted version.

## Funding

This work was supported by Janell and Joe Marek ‘57 Alzheimer’s Disease Research Fund (to NExT Dept.) and grant from the Alzheimer’s Association AARFD-16-440750 to KS.
